# Evaluation of a Novel Cuboid Hollow Fiber Hemodialyzer Design Using Computational Fluid Dynamics

**DOI:** 10.3390/membranes13010093

**Published:** 2023-01-11

**Authors:** Yating Xu, Umatheny Umatheva, Raja Ghosh

**Affiliations:** Department of Chemical Engineering, McMaster University, Hamilton, ON L8S 4L7, Canada

**Keywords:** hemodialyzer, computational fluid dynamics, cuboid hemodialyzer, flow distribution, shear rate, shear stress

## Abstract

Conventional hollow fiber hemodialyzers have a cylindrical shell-and-tube design. Due to their circular cross-section and radial flow distribution and collection in the headers, the flow of blood in the header as well as in the hollow fiber membranes is non-uniform. The creation of high shear stress and high shear rate zones or stagnation zones could result in problems, such as cell lysis and blood clotting. In this paper, a novel cuboid hemodialyzer design is proposed as an alternative to the conventional cylindrical hemodialyzer. The primary motivation behind the proposed design is to create uniform flow conditions and thereby minimize some of the above-mentioned adverse effects. The most salient feature of the proposed design is a cuboid shell within which the hollow fiber membrane bundle is potted. The lumen of the fibers is fed from one side using a flow distributor consisting of embedded primary and secondary channels, while the fibers are drained from the other side using a flow collector, which also has embedded primary and secondary channels. The flow characteristics of the lumen side of the cuboid hemodialyzer were compared with those of a conventional hemodialyzer based on computational fluid dynamics (CFD) simulations. The results of CFD simulations clearly indicated that the flow of liquid within the cuboid dialyzer was significantly more uniform. Consequently, the shear rate and shear stress were also more uniform. By adopting this new design, some of the problems associated with the conventional hemodialyzer design could potentially be addressed.

## 1. Introduction

**Figure 1:**
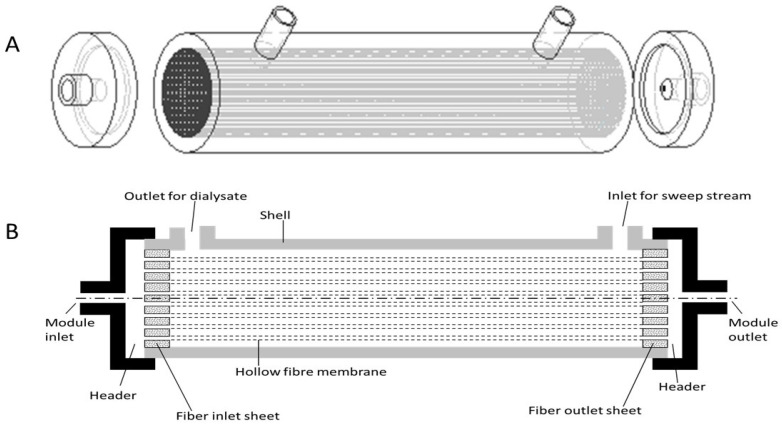
(**A**). Schematic drawing of a conventional hollow fiber membrane based hemodialyzer. (**B**) Section drawing of a conventional hollow fiber membrane based hemodialyzer.

End-stage renal disease (ESRD), which leads to loss of kidney function, affected more than 500,000 people in the US in 2021 [1]. For most patients, the chance for renal transplantation is usually low, since it is hard to find a matching donor. Therefore, hemodialysis is the frontline clinical procedure for treating kidney failures. It uses a device called a hemodialyzer to augment or substitute the functions of the patient’s kidney [2]. The patient’s blood goes through the hemodialyzer, and uremic toxins and excess fluid are removed through a membrane. Although different types of hemodialyzers have been developed over the years, most of those used today contain hollow fiber membranes [3,4,5,6]. A typical hollow fiber membrane based hemodialyzer has a shell-and-tube configuration, which consists of thousands of hollow fiber membranes with inner diameter in the range of 180–200 microns and wall thickness in the range of 30–40 microns, potted within a cylindrical housing (see Figure 1A) [3,4,5,6]. An inlet manifold (also called the arterial port) distributes the blood to the lumen of the hollow fiber membranes, while an outlet manifold (also called the venous port) collects the purified blood and directs it out of the device (see Figure 1B). The dialyzing fluid (also called the sweep stream) flows within the shell outside the hollow fiber membranes and removes any species that is transported through the membrane due to concentration difference driven diffusion (see Figure 1B). The hollow fiber inlet and outlet sheets are made of potting glue (e.g., epoxy resin), and these are analogous to the tube sheets present in shell-and-tube heat exchangers. Most of the species removed from blood by hemodialyzers are small molecules, such as urea and creatinine. Larger uremic toxins can be removed by hemofiltration, which combines diffusion and convection for enhancing solute transport through the membrane [7].

Hemodialyzers are being used with great clinical outcomes. However, as with anything, there is always room for improvement [7,8], and the quest for improvement should never cease. Hemodialyzers share a common design feature with some hollow fiber membrane modules used for applications such as bioprocessing and water treatment, i.e., the shell-and-tube configuration. Therefore, any improvement in the material and design of hemodialyzers could potentially lead to improvements in the hollow fiber membrane modules used for other applications, or vice versa. There has been a lot of research conducted on developing new material for making superior hollow fiber membranes for hemodialysis and other applications [9,10,11,12]. For instance, materials such as poly(lactic acid) [9], sulfonated polyether sulfone [10] have been developed, or hydrophilization of existing membranes using additives such as polyvinylpyrrolidone [11] and polyethylene glycol [12] has been proposed. While these approaches could lead to more hydrophilic, biocompatibilities or permeable dialysis membranes, some of the limitations of a hollow fiber hemodialyzer are due to its physical design [5,13,14,15,16,17,18,19]. Non-uniform flow has been flagged by several researchers as a major factor contributing toward poor performance of a hollow fiber membrane module [5,13,14]. Poor flow distribution could result in problems, such as poor clearance of toxins [15,16] and membrane fouling [17]. Non-uniform flow could also result in the creation of zones of high shear stress (or shear rate), or in some cases, stagnation, resulting in serious problems, such as lysis of blood cells [18] and the formation of blood clots [19].

Flow maldistribution in shell-and-tube configuration hollow fiber modules has been extensively examined [5,13,14,15,16,17]. Park and Chang [5] examined uneven flow distribution on the lumen side based on rigorous numerical methods and provided experimental proof for the same. They reported that the flow of fluid was highest in the fibers located at the center and that the shape of the header had a significant impact on flow behavior. In another study, Lemanski and Lipscomb [13] reported that the flow of fluid on the shell side of a typical hollow fiber membrane module was also non-uniform, and this had an impact on the separation efficiency, particularly on the clearance. Fouling due to non-uniform flow in hollow fiber membrane modules has been reported in the literature [17,20,21]. Researchers have also examined flow maldistribution issues pertinent to hemodialyzers [22,23,24,25,26,27,28,29,30,31,32,33,34]. Flow non-uniformity manifests itself right after the blood enters the header of the hemodialyzer [22]. The central location of the inlet and outlet for blood flow results in a flow bias that favors core (centrally located) fibers over peripheral fibers [23]. Ronco et al. [22] published an in vitro study where the difference in flow velocity of blood between the central and peripheral fibers of a hemodialyzer has been systematically examined. When the hematocrit value was 25%, the velocity difference was about 0.61 m/s, while when the hematocrit value was increased to 40% (which is within the normal ranges of adult males and females), the difference in blood velocity increased to about 1.6 m/s. Such uneven blood flow not only results in loss of efficiency (i.e., poor clearance of uremic toxins), but it also results in fiber-to-fiber variability and hemoconcentration [24,25]. Nowadays, high-flux hemodialyzers, which contain high-performance membranes, are favored, since they have the capability of removing middle to large size molecules. Flow maldistribution would impact such devices in a more significant way since high internal filtration (or back filtration) would create conditions suitable for severe membrane fouling [26]. The location of the ports on the shell side (see Figure 1) also contributes toward flow maldistribution outside the fibers through the creation of a stagnation zone, which decreases the effective diffusion rate [27].

Shear rate and the resultant shear stress are key factors in hemodialysis, as they not only affect membrane performance but impact the stability and viability of the blood cells being processed [28]. High shear stress could lead to rupture or damage of blood cells. As with flow rate, the shear rate and thereby the stress is highest at the center of the hemodialyzer due to the centrally fed, radially distributed flow arrangement in the header [29]. For smaller diameter or narrow flow channels, such as those in a hemodialyzer, the flow rate has a direct correlation with the shear stress [22,28,30]. Ronco et al. [22] demonstrated that the maximum wall shear rate in a hemodialyzer could be three times that of the minimum shear rate when operating under a 40% hematocrit condition. Under these conditions, the shear rate linearly correlated with shear stress. A more uniform flow within the hemodialyzer would therefore reduce the chances of cell lysis, which is known to occur under high shear stress conditions [31,32]. Blood clotting is another important factor that clinicians must consider when choosing a dialyzer [27]. Clotting in a hemodialyzer could result in severe problems, as the inner diameter of a hollow fiber membrane is quite small, and these are easily blocked [33]. Once clotting starts, the fiber blocking occurs very rapidly [19,33]. The common strategy to reduce clotting is to enhance biocompatibility by proper selection of the membrane and device material. However, material properties are not the only factors involved in clotting. Extremely low shear rate conditions that exist in stagnation zones within a hemodialyzer could also be responsible for blood clotting [22,30]. Clotting under non-uniform flow conditions could also reduce the effective surface area of the membrane and thereby lower the overall clearance of the hemodialyzer [27]. To minimize the possibility of thrombus formation and platelet adhesion and aggregation, the local flow velocities and shear rates must not be too low [34]. On the other hand, extremely high shear rates (>5000 s^−1^) would lead to problems, such as cell lysis, life-threatening hemorrhage or arterial occlusion [35].

In addition to improvements in membrane material, hemodialyzer performance could also be improved by addressing the following factors: design of the hemodialyzer device, proper selection of potting material and proper selection of operating conditions [8]. The packing density of the module, i.e., the number of fibers bundled in a device, would affect the fluid flow distribution in the module, both on the lumen and dialysate sides, thereby affecting dialyzer performance [17]. Several researchers have worked on optimizing the packing density in dialyzers for performance improvement. The average velocity of the dialysate on the shell side could be increased by increasing the packing density in the module [17]. There are several reports on the ways to increase fiber packing density and on how this affects dialyzer performance [36,37]. Other studies have focused on improving blood flow uniformity by using specially designed headers [26,36,38]. Studies have also reported the use of O-rings [23,39], baffles [40], spacer yarn [40,41] and wavy fibers [27] for improving flow uniformity. However, it is worth noting that the basic design of all these devices remains the same, i.e., each consists of a cylindrical hollow fiber membrane bundle housed within a cylindrical shell, with the distribution and collection headers having a circular cross-section.

**Figure 2:**
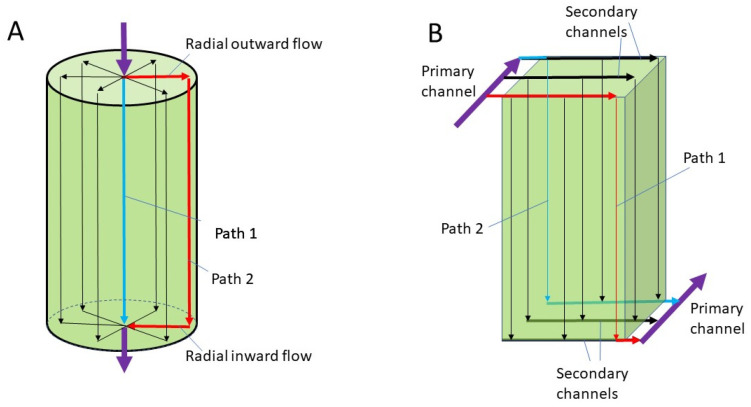
(**A**) Idealized flow paths in a conventional cylindrical packed bed. (**B**) Idealized flow paths in a z^2^ cuboid packed bed.

In this paper, we propose a radically different design for a hollow fiber membrane based hemodialyzer. This new design is based on the cuboid chromatography devices, which were developed in our research group as alternatives to conventional cylindrical chromatography columns [42,43,44,45]. The basic hypothesis behind the cuboid chromatography devices is that the cuboid shape is a better option than the cylindrical shape in terms of flow uniformity for packed beds having large cross-sectional areas. Conventional process chromatography columns have serious flow maldistribution problems, primarily due to two reasons. Firstly, due to their cylindrical shape and axial feeding and draining, the path lengths vary quite significantly, i.e., the flow path along the axis is significantly shorter than those closer to the wall of the column (see Figure 2A) [42]. Secondly, conventional columns rely on radially outward and radially inward flow in the inlet and outlet headers for flow distribution and collection, respectively. It has been amply demonstrated through first principle mathematical analysis and computational fluid dynamic (CFD) simulations that the radial velocities in the header decrease exponentially in a radially outward direction [42,46,47]. Therefore, a solute molecule traveling down the axis of a conventional column not only travels a shorter distance than a solute traveling down the wall of the column, but it also travels at a significantly higher average velocity than the latter (see Figure 2A) [42,46,47]. The z^2^ flow distribution and collection system, which has successfully been used in resin and membrane-based chromatography, addresses the problem of flow maldistribution by reformatting the separation media (resin or membrane) into a cuboid shape [42,43,44,45,46,47]. The cuboid separation media are then fed from one side using a combination of a primary and a set of secondary channels and drained from the other side of the device using a complimentary set of primary and secondary channels (see Figure 2B). The cuboid shape of the media, coupled with the specific flow arrangement, makes the path lengths within the packed bed and the average velocities along these more uniform. This results in a more uniform flow of the mobile phase through the chromatographic media, leading to significant improvement in the quality of separation [42,43,44,45].

The above explanation for flow maldistribution in a wide column could be extended to explain flow maldistribution in a conventional cylindrical hemodialyzer, i.e., blood traveling down a fiber located along the axis of a cylindrical hemodialyzer not only travels a shorter distance than blood traveling down a fiber located close to the wall, but it also travels at a significantly higher average velocity than in the latter. Therefore, it is tempting to think that by reformatting the cylindrical hollow fiber membrane bundle to one with a cuboid shape and by employing primary and secondary channels similar to those in the cuboid chromatography devices, better flow uniformity could be achieved in a hemodialyzer. In this paper, we explore this idea based on computational fluid dynamics (CFD), which is a powerful tool that allows preliminary feasibility testing of design ideas and concepts before embarking on the long path of real physical design development. CFD also makes it possible to determine the expected values for flow velocity, pressure, shear rate, shear stress in the different sections of the flow path, based on which physical prototype devices could be designed.

## 2. Materials and Methods

### 2.1. Design Overview

**Figure 3:**
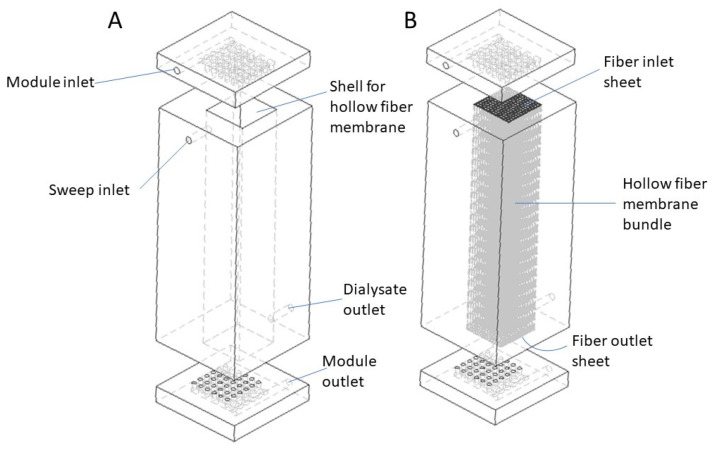
(**A**) Schematic diagram of the cuboid shell and the flow distribution and collection plates of the proposed cuboid hemodialyzer. (**B**) Proposed cuboid hemodialyzer with hollow fiber membrane in place.

Figure 3 shows the schematic drawings of the proposed cuboid dialyzer. The overall shape of the cuboid dialyzer is different from that of a conventional cylindrical hemodialyzer. Figure 3A shows the empty cuboid shell and the flow distributor and collector plates. The module inlet on the flow distributor plate serves as the arterial port, while the module outlet on the collector plate serves as the venous port. The sweep inlet and the dialysate outlet are also clearly indicated in Figure 3A. Unlike in a conventional cylindrical hemodialyzer, where these are located on the same side (see Figure 1), in the cuboid hemodialyzer, these are located at space diagonally opposite ends. The motivation for doing so is to ensure uniform flow of dialysate within the shell space outside the fibers. However, this intuitive hypothesis is not tested or verified in the current study, as it primarily focuses on flow on the lumen side, i.e., the side where the blood flows. Figure 3B shows the position of the hollow fiber membrane bundle within the cuboid hemodialyzer. The overall shape of the hollow fiber bundle is also cuboid, and it fits in the cuboid shaped hollow within the shell. In a physical cuboid hemodialyzer, the fiber bundle would be potted using an appropriate potting resin, such as epoxy glue, to generate the fiber inlet sheet on one side and a fiber outlet sheet on the other side.

**Figure 4:**
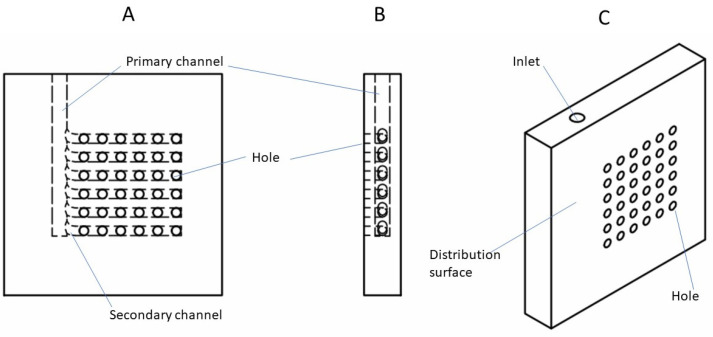
(**A**) Top view of flow distribution plate. (**B**) Side view of flow distribution plate. (**C**) Isometric view of flow distribution plate.

Figure 4 shows the drawings of the flow distribution plate for blood entering the lumen of the fiber bundle from the arterial port. The blood is directed to a primary channel, from which a set of secondary channels branch off sequentially. The primary and secondary channels are embedded within the flow distribution plate. Each of the secondary channels is further connected to a distribution surface through a series of holes (see Figure 4). The liquids flowing through these holes are further distributed to the bundle of hollow fiber membranes potted within a cuboid shell through the fiber inlet sheet (see Figure 3B). A small gap between the distribution surface and the fiber inlet sheet serves as the inlet header. The collection plate that drains the blood from the fiber bundle (through the fiber outlet sheet) to the venous port also has a set of secondary channels and a primary channel, which ultimately connects to the outlet (the venous port). As with the inlet side, the secondary channels are connected to a collection surface through a series of holes. A small gap between the fiber outlet sheet and the collection surface serves as the outlet header. The channels in the collection plate have the same dimensions and geometry as their corresponding counterparts in the flow distribution plates, but the arrangements of the channels in the two plates are complimentary, i.e., the plates are fabricated as mirror images of each other (see Figure 3). Such arrangement of the flow channels in the two plates is necessary to ensure that the flow within the device is uniform [42,46,47]. As can be seen in Figure 3, the inlet and outlet for blood in the cuboid hemodialyzer are located space diagonally with respect to each other.

In this study, two cuboid hemodialyzers (cuboid 1 and cuboid 2) are compared with a conventional cylindrical hemodialyzer (designated conventional) by CFD simulations. The comparison is made based on the flow velocity, shear rate and shear stress in the flow field and the tracer flow through these devices. The inlet radius for all these devices was 2 mm (based on standard dimension used in commercially available hemodialyzers) [46]. The dimensions of the conventional hemodialyzer were also based on those of a commercially available device [46]. The radius of the primary channels in the two cuboid hemodialyzers was the same, i.e., 2 mm, while the secondary channel radii for cuboid 1 and cuboid 2 were 1.0 mm and 1.25 mm, respectively. Each cuboid device had six secondary channels in each of its collection or distribution plates. The number of holes connecting each of the secondary channels to the respective header was 6. Therefore, each plate had 36 holes for flow of blood from the plate to the fiber bundle on the inlet side and for flow from the fiber bundle to the collecting plate on the outlet side. The length and number of hollow fiber membranes in all these devices were kept the same. Additionally, the hollow fibers had the same inner and outer diameter. Due to the limitation in computing power and time, the number of fibers was limited to 900. For the cuboid hemodialyzers, there was one hole in each plate for every 25 hollow fiber membranes. Therefore, the holes were located such that each could service 25 hollow fiber membranes arranged in a 5 × 5 array. Table 1 summarizes the design information for the 3 devices simulated in this study.

### 2.2. Model Development

COMSOL Multiphysics 6.0 (COMSOL, Inc., Burlington, MA, USA) was used to carry out the computational fluid dynamic simulations for this study. Two physics packages were used: Laminar Flow for steady-state simulation and Transport of Diluted Species for time-dependent tracer studies. The finite element method (FEM) was applied, physics-controlled mesh was used, and the element sizes were coarse in all the models.

#### 2.2.1. Assumptions

The following assumptions were made to simplify the model:

(1) Blood is a viscous and incompressible fluid [47,48]. Blood shows significant non- Newtonian properties, and the plan is to examine the effects of non-idealities resulting from non-Newtonian flow in future studies.
(2) The lumen of the membranes is always filled with liquid.
(3) There is no clotting in the bloodstream.
(4) Fully developed laminar flow is assumed within the lumen [49].
(5) Since this work focuses on fluid flow characteristics in the headers and within the membrane lumen, the transport of species through the membrane wall is not considered. The hollow fiber is considered to be a cylindrical tube with impermeable wall. Blood is transported along the length of the hollow fiber without any change in composition [50].
(6) No slip conditions exist at the wall [51].
(7) Although the neighboring fibers in a real hemodialyzer are randomly spaced due to the nature of the manufacturing process, the fibers in the simulated devices are considered to be equally spaced according to a specific pattern.

#### 2.2.2. Numerical Method

At each “mesh node” in the CFD models, the mass balance, i.e., the continuity Equation (1), and momentum balance, i.e., the Navier–Stokes Equation (2), were established and solved to determine the nature of fluid flow in the conventional and cuboid hemodialyzer.

(1) $$\frac{\partial \rho }{\partial t}+\rho \nabla \cdot u=0$$

(2) $$\rho \left(\frac{\partial u}{\partial t}\right)+\rho \left(u\cdot \nabla \right)u=-\nabla P+\nabla \left(\mu \left(\nabla u+{\left(\nabla u\right)}^{T}\right)-\frac{2}{3}\mu \left(\nabla \cdot u\right)Ι\right)+F$$

For fully developed laminar, incompressible and viscous flow with no-slip conditions, the Navier–Stokes equations can be simplified to Equation (3) (time-dependent) and Equation (4) (steady-state):

(3) $$\rho \left(\frac{\partial u}{\partial t}\right)+\rho \left(u\cdot \nabla \right)u=-\nabla P+\nabla \left(\mu \left(\nabla u+{\left(\nabla u\right)}^{T}\right)\right)+F$$

(4) $$\rho \left(u\cdot \nabla \right)u=-\nabla P+\nabla \left(\mu \left(\nabla u+{\left(\nabla u\right)}^{T}\right)\right)+F$$

As the density of an incompressible fluid would not change with time, the continuity equation can be simplified to Equation (5) below:

(5) ∇·u=0

The parameters used in the CFD simulations are summarized in Table 2.

In addition to steady-state simulations for velocity, shear rate and shear stress, dynamic tracer tests were performed using the Transport of Diluted Species physics package to compare solute dispersion in the hemodialyzers. Sodium chloride solution (0.8 mol/L) was introduced at the device inlet as a pulse function. The volume of the tracer used in these simulations was 5% of the total internal volume of the flow path.

#### 2.2.3. Limitations

In CFD simulations, there is a trade-off between the precision of the model and the computational cost (i.e., time of the process, size of the file and memory requirement) [52,53]. In this study, which involved many simulations, physics-controlled mesh was created in coarse element size, which was considered sufficient for obtaining satisfactory results. The nodes were discrete instead of being continuous. In this study, the length to radius ratio of a hollow fiber was higher than 1200. Therefore, the circular cross-section of the membrane had to be approximated as a square. However, this was performed consistently for all membrane fibers, and this is therefore not expected to affect the results of the device-to-device comparison. The radius of the hollow fiber membranes used in the simulation was 4 times that of a typical fiber used in a real clinical hemodialyzer. This had to be done, as fine mesh simulation would otherwise be required, in which case, the simulations would continue indefinitely. Due to the larger radius of the hollow fiber membrane, the number of fibers had to be decreased for that typically used (a few 1000s) to 900.

## 3. Results and Discussion

Flow velocity, shear rate and shear stress data for the three devices (conventional and the two cuboids) were obtained through steady-state simulations, while a time variant study was used to simulate the tracer experiments.

### 3.1. Flow Distribution

**Figure 5:**
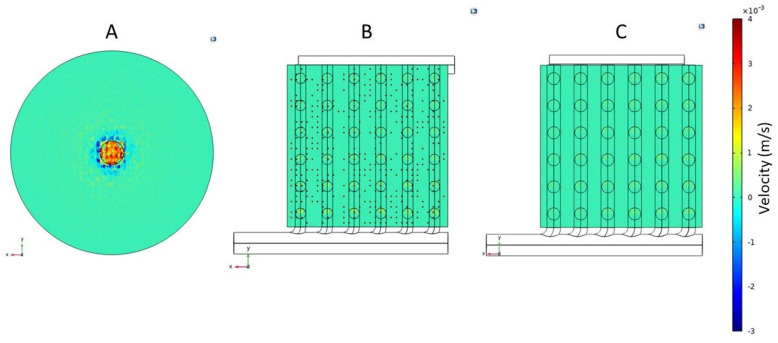
Velocity vector (z-direction) profiles for the fiber inlet plane of (**A**) Conventional cylindrical hemodialyzer. (**B**) Cuboid hemodialyzer 1. (**C**) Cuboid hemodialyzer 2.

Figure 5 shows the velocity vectors in the direction of the fluid flow (i.e., the z-direction) at the entrance of the hollow fiber membranes (i.e., at the surface of the fiber inlet sheet) for the conventional and the two cuboid hemodialyzers. A positive velocity vector indicated that the flow at that point was in the same direction as the overall blood flow in the hemodialyzer, while a negative velocity vector indicated backflow at that location. As can be seen in Figure 5A, there was considerable variation in velocity at the fiber entrance of the conventional hemodialyzer. Very close to the center, corresponding to the region directly adjoining the device inlet, the velocity at the fiber entrance was very high, almost an order of magnitude higher than that in the vast majority of the fibers located further away from the center. This was due to the jetting effect of blood flowing in through the inlet. Interestingly, just beyond the zone of high velocity, there was a zone where the velocity vectors were negative, i.e., these were directed opposite to the direction of flow. The impingement of the inflowing blood on the impermeable portion of the fiber inlet sheet, i.e., where the potting resin is present, would result in significant flow in the opposite direction (relative to the direction of flow within the hollow fibers). Another factor likely responsible for the negative velocity is the abrupt change in direction of fluid as it enters the header from the device inlet. The fluid enters through a tube with a radius of 2 mm into a shallow disc-shaped space where it has to transition from axial flow to radial flow. Such high-velocity radial flow could result in localized zones of low pressure at the fiber entrance, leading to the drawing of liquid from the fiber in the opposite direction. However, beyond this “negative velocity” zone, the fiber entrance velocity was more or less uniform. Figure 5B,C shows the fiber inlet velocity vectors for the two cuboid hemodialyzers. While some isolated high-velocity vectors can be seen at the fiber inlets for cuboid 1, the velocity vectors for cuboid 2 were very uniform. This difference between the two cuboid devices could be explained based on the difference in radii of the secondary channels and connecting holes in these devices. The smaller secondary channel radius and hole radius of cuboid 1 would result in blood flowing through these at higher velocities than in cuboid 2. Therefore, any jetting effect in the header would be more pronounced in cuboid 1. There were no negative fiber inlet velocity vectors in either cuboid 1 or cuboid 2, which indicated that there was no or negligible back mixing in these devices. These results provide the first indication that the cuboid hemodialyzers were better from a flow uniformity perspective. These results also indicate that the dimension of the flow channels in the cuboid hemodialyzers had a significant impact on the velocity of flow at the fiber inlets, i.e., there was room and need for optimization of the channel dimension. The fully developed flow of liquid within the lumen of the hollow fiber was laminar, with the average Reynolds number being less than 10.

**Figure 6:**
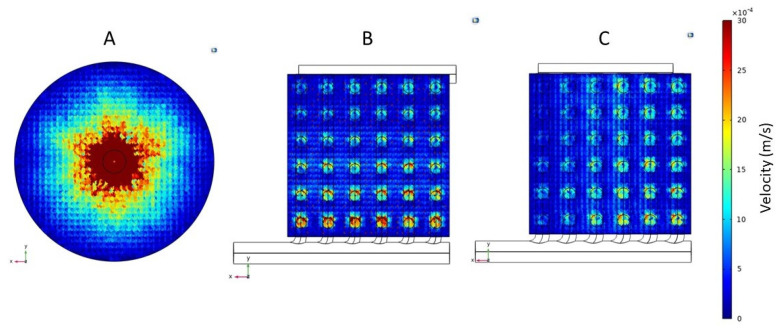
Overall velocity profiles for the fiber inlet plane of (**A**) Conventional cylindrical hemodialyzer. (**B**) Cuboid hemodialyzer 1. (**C**) Cuboid hemodialyzer 2.

Figure 6 shows the overall velocity at the inlet plane of the hollow fiber bundle in the three devices (i.e., the conventional and the two cuboids). The overall velocity profile combines velocity vectors along the x-, y- and z-directions and presents the magnitude of the resultant, without providing the direction of the resultant vector. The overall velocity profile for the conventional hemodialyzer (see Figure 6A) indicated a fairly large high overall velocity zone centered around the device inlet. A comparison with the corresponding z-direction velocity vector profile (see Figure 5A) shows that the overall velocity is overwhelmingly dominated by the non-z-direction velocity components, which in this case would be in a radially outward direction. The basic design feature of the conventional cylindrical hemodialyzer is based on radial flow distribution in the header, and this implies that the velocity in the radial direction would be very high closer to the inlet and would drastically diminish as we approach the periphery. This is consistent with that observed in the headers of centrally fed cylindrical chromatography columns [42,46,47]. By using the combination of primary and secondary channels, the blood could be more gently and uniformly distributed to the fiber bundle without the axial jetting and the radial flow observed with the conventional cylindrical hemodialyzer. In other words, the flow in the cuboid devices was more ordered (with less turbulence), and the energy associated with the inflowing blood was more efficiently translated to flow into and within the fibers. Between the two cuboid devices, cuboid 2 had a significantly more uniform flow, which indicated once again that the dimensions of the secondary channels and the connecting hole were important design variables, which would need to be optimized to design an efficient cuboid hemodialyzer.

### 3.2. Shear Rate and Stress

**Figure 7:**
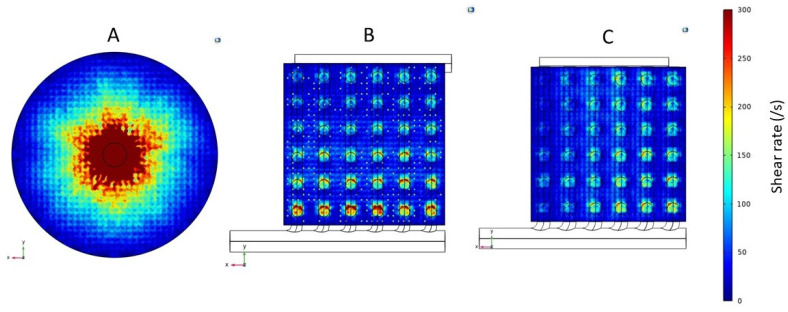
Shear rate profiles for the fiber inlet plane of (**A**) Conventional cylindrical hemodialyzer. (**B**) Cuboid hemodialyzer 1. (**C**) Cuboid hemodialyzer 2.

**Figure 8:**
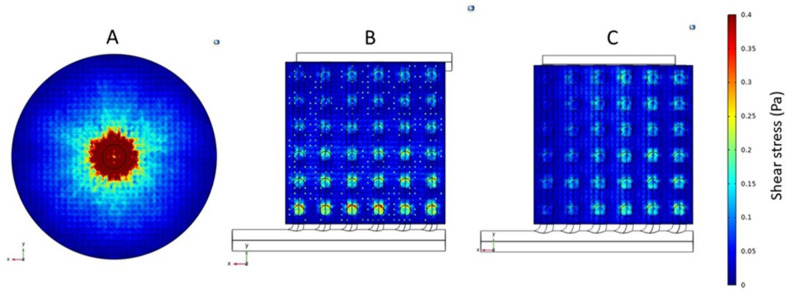
Shear stress profiles for the fiber inlet plane of (**A**) Conventional cylindrical hemodialyzer. (**B**) Cuboid hemodialyzer 1. (**C**) Cuboid hemodialyzer 2.

The results discussed in the preceding paragraphs clearly showed that the flow non-uniformity in the conventional cylindrical hemodialyzer was primarily due to the dependence on radial flow in the header for flow distribution. In particular, the very high radial velocity close to the central region of the header on the inlet side would likely manifest itself in terms of high shear rate and shear stress in this region. Figure 7 shows the shear rate profiles, and Figure 8 shows the shear stress profiles at the fiber inlet plane for the three devices (i.e., the conventional and the two cuboids). As expected, the shear rate and shear stress profiles in the three devices mirror their corresponding overall velocity profiles. The conventional cylindrical hemodialyzer had a central zone of extremely high shear rate and shear stress. By comparison, the shear rate and shear stress in both cuboid devices were more uniform, and more importantly, these devices did not have zones of extremely high shear rate or shear stress, where blood cells could potentially be damaged.

### 3.3. Tracer Study

**Figure 9:**
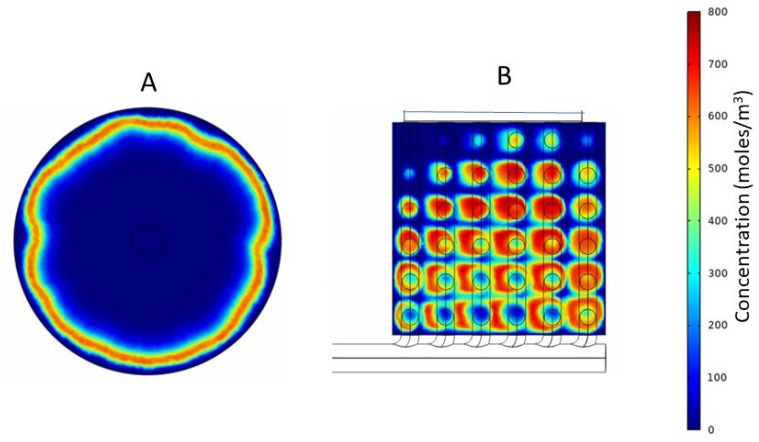
Tracer concentration profiles at the mid-section plane of the header of (**A**) Conventional cylindrical hemodialyzer. (**B**) Cuboid hemodialyzer 2.

Tracer studies are useful for determining residence time distribution in flow through devices. A pulse of the tracer is introduced along with the flowing feed, and the extent to which the solute in the pulse is dispersed during its transit through the device is determined by measuring its concentration at the outlet as a function of time (or effluent volume), i.e., the sharper the solute peak, the narrower the residence time distribution. The residence time distribution is a useful tool for measuring flow uniformity (or non-uniformity). The tracer flow simulations were carried out using the conventional cylindrical hemodialyzer and cuboid 2, which was the better of the two cuboid devices. The tracer solution used was 800 mol/m^3^ sodium chloride in water. The volume of sodium chloride solution injected was 5% of the total internal volume of the flow path, and the flow rate was the same as the operational flow rate used in the previous experiments. CFD allows visualization of the transit of a tracer pulse through a device. Such visualization, which is based on a color coding for concentration of the tracer within the device, is useful, as it provides information, such as the location of dead zones and reasons for flow maldistribution, based on which, a device could be redesigned for better performance. It also allows qualitative comparison of the flow characteristics of different devices. Figure 9 shows the solute (sodium chloride) concentration profile at the mid-section of the headers of the two devices (conventional and cuboid 2), 0.8 s after tracer injection. The solute profile observed in the conventional hemodialyzer (see Figure 9A) indicated that at a time when solute was still present near the periphery, solute molecules traversing through the fibers located closer to the center had already entered the fiber and were hence no longer visible. Therefore, the solute residence time distribution of the conventional cylindrical dialyzer could be expected to be broad. With cuboid 2 (see Figure 9B), the tracer solute could be observed throughout the header at the snapshot time. However, due to the sequential branching of the secondary channels from the primary channel, some location-dependent differences in tracer concentration could be expected in the headers of the cuboid devices. For instance (see Figure 9B), when the solute had started disappearing in the region adjacent to the leftmost hole in the bottom row (as evident from the central discoloration), a significant amount of solute could be seen in the regions adjacent to the rightmost hole in the bottom row. Such lead and lag in concentration is compensated at the outlet side where the secondary channels and the primary channel are arranged in a complementary fashion with respect to the inlet side (see Figure 3).

**Figure 10:**
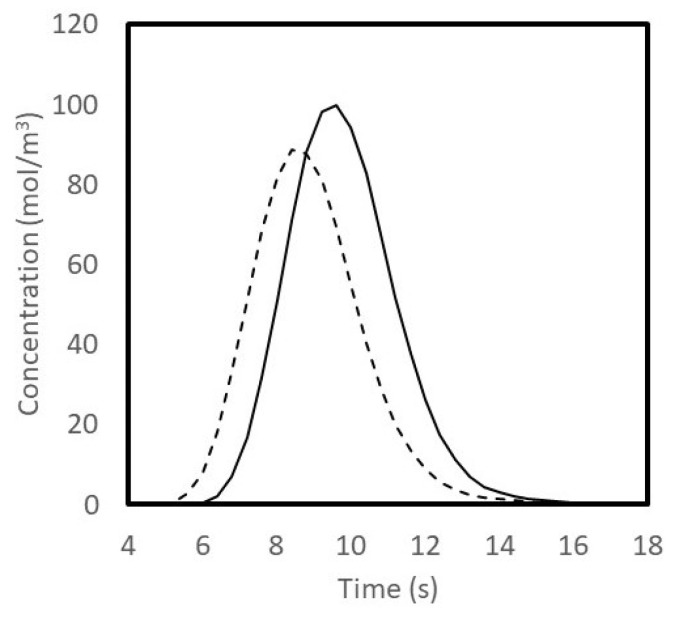
Tracer (sodium chloride) concentration peaks for conventional cylindrical hemodialyzer (dashed line) and cuboid hemodialyzer 2 (solid line).

Figure 10 shows the tracer peaks obtained with the conventional cylindrical hemodialyzer and cuboid 2. As expected from the results shown in Figure 9, the tracer peak obtained with cuboid 2 was sharper and taller than that obtained with the conventional hemodialyzer. The tracer peaks shown in Figure 10 are indicative of the solute retention time distribution within these devices. The sharper and taller peak obtained with the cuboid hemodialyzer indicates that the flow of blood was more uniform within it. One of the rules of thumb for obtaining a narrow residence time distribution is to maintain a hierarchy in hydraulic resistance (i.e., highest resistance in the fibers, lower resistances in the secondary channels and lowest resistance in the primary channel) [42,44].

The simulation results discussed in the paper indicate that the cuboid hemodialyzer could potentially solve some of the problems associated with the conventional cylindrical hemodialyzer. The flow in a cuboid device is expected to be more uniform, and the high shear rate and shear stress zones seen in the conventional hemodialyzer could potentially be eliminated. However, the “proof of the pudding is in the eating”, and therefore, any definitive claims could only be made based on real experiments carried out using physical devices. The prototype cuboid hemodialyzer device is currently being fabricated, and we hope to carry out the comparison experiments soon. The cuboid hemodialyzer design proposed in this paper could also be adapted and adopted for designing other types of hollow fiber membrane devices, such as those used for industrial dialysis [14], hollow fiber membrane contactors for extraction [54] and crystallization [55], ultrafiltration [56], microfiltration [57] and as membrane bioreactors [58]. The design of the cuboid hemodialyzer could also be adopted and adapted for other process engineering devices, such as heat exchangers and monolith reactors. In future simulation studies, the transport of permeable species through the hollow fiber membranes will be modeled based on multi-scale computational models for hollow fiber hemodialysis reported in the literature [16,59]. The current study focuses on flow in the lumen side of a hemodialyzer. The logical next step would be to look at how flow in the shell side influences hemodialyzer performance. It is anticipated that the positioning of the shell side ports could have a significant impact on separation performance.

## 4. Conclusions

The cuboid hemodialyzer design proposed in this paper could potentially solve some of the problems associated with the conventional cylindrical hemodialyzer. Simulation studies showed that there is significant back mixing in the header of the conventional hemodialyzer, particularly close to the inlet. This resulted in regions of high shear rate and high shear stress, which could be detrimental to the blood cells. It was shown based on computational simulations that the overall flow of blood in the cuboid hemodialyzer was significantly more uniform. The flow of blood in and out of the proposed cuboid hemodialyzer is based on complimentary sets of primary and secondary channels. Due to such flow arrangement, jetting of blood from the device inlet into the header was minimized. Consequently, the flow in the fiber bundle of the cuboid hemodialyzer was more uniform, with no observable back mixing. Additionally, the high shear rate and shear stress zones could be avoided. While the simulations do seem to suggest that the proposed cuboid hemodialyzer would be better than the conventional hemodialyzer in several respects, real experiments carried out using physical cuboid hemodialyzers fabricated based on the proposed design would be necessary before firm claims could be made.

## Figures and Tables

**Table 1:** Design information for hemodialyzers used in the CFD simulation study.

Parameter	Value
Depth of the header (manifold)	0.001 m
Length of hollow fiber membrane	0.245 m
Effective length of membranes (excluding dimension lost in tube sheet)	0.230 m
Inner radius of arterial/venous/dialysate port	0.002 m
Inner radius of the membranes	2.00 × 10^−4^ m
Outer radius of the membrane	2.45 × 10^−4^ m
Number of hollow fiber membranes	900
Radius of header (manifold) in conventional	0.017 m
Inner radius of shell of conventional	0.017 m
Outer length of conventional	0.030 m
Outer length of cuboid (both cuboids)	0.030 m
Radius of primary channel (both cuboids)	0.00200 m
Radius of secondary channels (cuboid 1)	0.00100 m
Radius of hole (cuboid 1)	0.00100 m
Radius of secondary channels (cuboid 2)	0.00125 m
Radius of hole (cuboid 2)	0.00125 m

**Table 2:** Parameters used for CFD simulations.

Parameter	Value
Inlet volumetric flow rate	2.5 × 10^−6^ (m^3^/s)
Temperature	298.15 Kelvin
Concentration of the tracer solution (NaCl)	0.8 (mol/L)
Diffusion coefficient of NaCl in water	2.9 × 10^−9^ (m^2^/s)

## Data Availability

Data will be made available on reasonable request.

## Nomenclature

**Symbol**	**Parameter**
*ρ*	Fluid density (kg/m^3^)
*t*	Time (s)
*u*	Fluid velocity field (m/s)
*P*	Pressure (Pa)
*μ*	Fluid dynamic viscosity (Pa s)
*I*	Identity matrix (-)
*F*	External forces (Pa)
